# Meeting the estimated daily optimal standardized ileal digestible lysine-to-net energy ratios for first and second parity lactating sows improved piglet growth rates

**DOI:** 10.1093/tas/txaf070

**Published:** 2025-05-26

**Authors:** Nicole L Gregory, Lee-Anne Huber

**Affiliations:** Department of Animal Biosciences, University of Guelph, Guelph, ON, N1G 2W1, Canada; Department of Animal Biosciences, University of Guelph, Guelph, ON, N1G 2W1, Canada

**Keywords:** lactation, net energy, precision feeding, sow, standardized ileal digestible lysine

## Abstract

One hundred three sows (52 first parity and 51 second parity) were used to evaluate the effect of meeting estimated daily optimal standardized ileal digestible (**SID**) Lys-to-net energy (**NE**) ratios throughout a 21-day lactation on piglet growth performance and sow body mobilization. A 2 × 2 factorial experimental design with the variables of parity and feeding program was used (n = 25 or 26). First and second parity sows were fed either a static feeding curve providing 3.9 g SID Lys/Mcal NE throughout the entirety of lactation (**CON**) or a dynamic feeding program that met estimated daily optimal SID Lys-to-NE ratios during lactation for maternal nitrogen retention (first parity sows; ranging from 5.48 to 4.95 g SID Lys/Mcal NE on days 1 and 20, respectively) or milk nitrogen output (second parity sows; ranging from 3.12 to 4.68 g SID Lys/Mcal NE on days 1 and 20, respectively; **PRE**). Weekly optimal SID Lys-to-NE ratios were determined for lactating primiparous and multiparous sows in previous studies, whereby only maternal nitrogen retention and only milk nitrogen output were influenced by SID Lys-to-NE ratio for primiparous and multiparous sows, respectively. Performance outcomes were not influenced by the interactive effect of feeding program and parity. Average daily feed intake did not differ between the CON and PRE feeding program, while second parity sows consumed more feed than first parity sows (Parity; *P* < 0.05). No differences were observed for maternal BW loss between the CON and PRE feeding programs, while second parity sows lost less BW than first parity sows (Parity; *P* < 0.05). Sows on the PRE feeding program tended to lose less backfat depth (**BF**) than sows on the CON program, regardless of parity (Feeding program; *P* = 0.094) and second parity sows lost less BF than first parity sows, regardless of feeding program (Parity; *P* < 0.05). Overall piglet average daily gain (**ADG**; 256 vs 246 ± 6 g) and piglet BW at weaning (6.45 vs 6.19 ± 0.17 kg) were greater for sows that received the PRE compared to the CON feeding program, regardless of parity (Feeding program; *P* < 0.05), with second parity sows having greater piglet BW and ADG than first parity sows, regardless of feeding program (Parity; *P* < 0.05). Therefore, providing a dynamic feeding program to meet estimated daily optimal SID Lys-to-NE ratios during lactation improved piglet growth performance without increasing maternal body weight losses in first and second parity sows.

## Teaser

Providing a feeding program that met estimated daily optimal lysine-to-net energy ratios for first and second parity lactating sows improved piglet pre-weaning growth performance. Implementation of dynamic feeding programs could provide useful in meeting parity-specific nutrient and energy needs in lactation.

## INTRODUCTION

Lactation creates substantial nutrient and energy demands for sows in order to produce adequate milk to support large and robust piglets by weaning, while maintaining sufficient maternal body condition to minimize nonproductive days. Milk is the main source of nutrients for piglets and larger litter sizes increase the demands for Lys and energy, with each piglet magnifying Lys requirements by approximately 1.7 g standardized ileal digestible (**SID**) Lys per day (Kim et al., 2001). Additionally, SID Lys and energy requirements increase in a dynamic fashion following the milk production curve (Feyera and Theil, 2017; Gauthier et al., 2019). Moreover, sow feed intake is inadequate relative to nutrient and energy outputs in milk, which is exacerbated for primiparous sows that have a smaller gut but greater nutrient and energy requirements for maternal growth versus mature sows (NRC, 2012). If sows are unable to consume sufficient feed during lactation, increased body tissue mobilization will occur to overcome intake deficiencies, which can be detrimental for lactation performance as well as rebreeding rates and subsequent litter size (Schenkel et al., 2010; Hoving et al., 2011; as reviewed by Tokach et al., 2019). Therefore, the dietary supply of nutrients and energy are key factors for supporting performance of lactating sows.

In practice, primiparous and multiparous sows are typically fed the same diet with static nutrient composition and ad libitum feed allowance to maximize nutrient intake throughout the lactation period, despite differing feed intake capacities and nutrient requirements among parities. For example, in multiparous sows (average parity 3.1), the SID Lys-to-net energy (**NE**) ratios necessary to maximize piglet growth rates in each week of lactation were found to be dynamic, while maternal nitrogen retention was not affected by the SID Lys-to-NE ratio (Watzeck and Huber, 2024). Conversely, in primiparous sows, the SID Lys-to-NE ratios necessary to minimize maternal tissue mobilization increased in each week of lactation, while piglet growth rates were not affected by the SID Lys-to-NE ratio (Gregory and Huber, 2025), indicating differing nutrient partitioning priorities for first and multiparous sows. Thus, there is potential to utilize such feeding systems to precisely meet the dynamic optimal SID Lys-to-NE ratios for both multiparous and primiparous sows throughout lactation by using basal diets with extreme SID Lys-to-NE ratios.

It was hypothesized that feeding programs with (daily) SID Lys-to-NE ratios designed to optimize maternal nitrogen retention for first parity sows (ranging from 5.48 to 4.95 g SID Lys/Mcal NE on days 1 and 20, respectively) and milk nitrogen output for second parity sows (ranging from 3.12 to 4.68 g SID Lys/Mcal NE on days 1 and 20, respectively) will improve piglet growth performance by minimizing maternal tissue mobilization. The objective of the current study was to implement and assess a precision feeding program that matched our previously estimated daily optimal SID Lys-to-NE ratios throughout lactation on piglet growth and sow lactation performance for first and second parity sows.

## MATERIALS AND METHODS

The study was conducted at the Ontario Swine Research Center (Elora, ON, Canada). All protocols and procedures were approved by the University of Guelph animal care committee (animal utilization protocol 4988) and follow the Canadian Animal Care Committee guidelines (CCAC, 2009).

### Animals and Housing

One hundred three PIC Camborough [52 first parity and 51 second parity sows (Pig Improvement Company, Winnipeg, MB, Canada)] were enrolled over 4 monthly farrowing batches (blocks). Only first and second parity sows were available at the research facilities due to a recent repopulation. Sows were moved into the farrowing room on day 110 of gestation into crates equipped with a creep area, a heat mat, and an automatic sow feeding system with feed blending capabilities (Gestal Quattro Pro, JYGA, QC, Canada). Sows were fed a blend of two basal diets to achieve a standard SID Lys-to-NE ratio of 3.91 g SID Lys/Mcal NE, which was provided at 2 kg per day prior to farrowing. Immediately after farrowing, and prior to implementing the respective feeding programs, litter characteristics were recorded (born alive, stillborn, mummified, and litter birth weight). Within 24 hours of birth, litters were standardized to 13 ± 1 piglets prior to assignment of lactation feeding program and ensuring no more than one piglet per teat. Thereafter, sows were assigned to feeding programs (described below) and received the respective feeding program until weaning, which occurred on day 20 ± 0.4 of lactation. Piglets were processed within 24 hours of birth following standard farm protocol (ear notched, teeth clipping, tail docking) and on day 5, piglets received iron injections and males were surgically castrated. Piglets were not provided with creep feed.

### Diets

**Figure 1.**
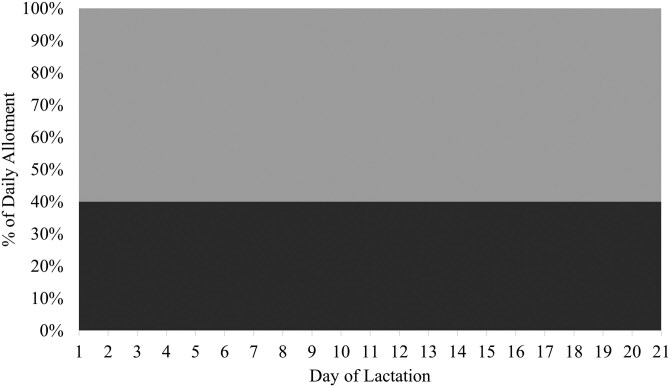
Two basal diets were formulated to have extreme standardized ileal digestible (SID) Lys-to-net energy (NE) ratios: 2.85 g SID Lys/Mcal NE (light gray) and 5.50 g SID Lys/Mcal NE (dark gray). A static blend was created to provide a ratio of 3.91 g SID Lys/Mcal NE to mimic the SID Lys-to-NE ratio of a commercial lactation diet fed to first and second parity sows throughout a 21-day lactation period.

**Figure 2.**
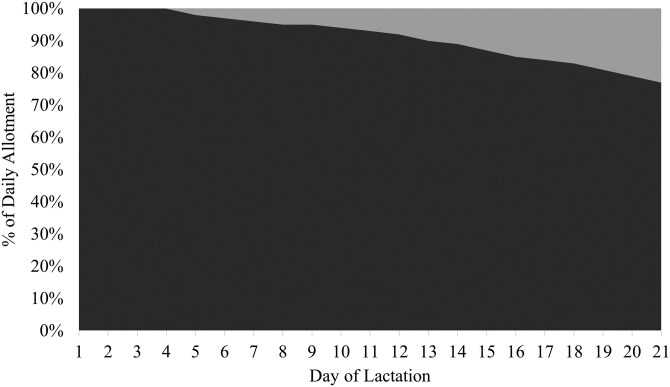
Two basal diets were formulated to have extreme standardized ileal digestible (SID) Lys-to-net energy (NE) ratios: 2.85 g SID Lys/Mcal NE diet (light gray) and 5.50 g SID Lys/Mcal NE diet (dark gray). The basal diets were used to create a dynamic blend that provided a daily ratio of SID Lys-to-NE to optimize maternal nitrogen retention for first parity sows throughout a 21-day lactation period.

**Figure 3.**
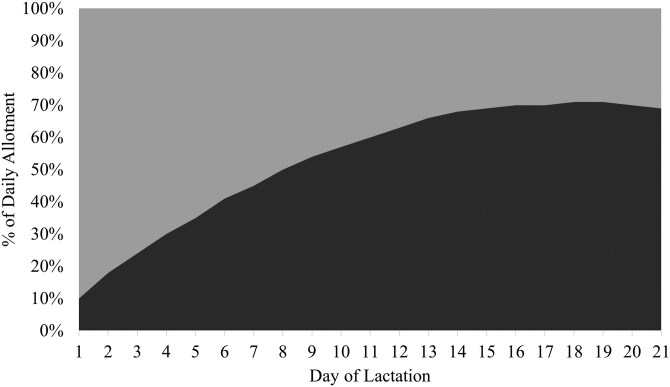
Two basal diets were formulated to have extreme standardized ileal digestible (SID) Lys-to-net energy (NE) ratios: 2.85 g SID Lys/Mcal NE diet (light gray) and 5.50 g SID Lys/Mcal NE diet (dark gray). The basal diets were used to create a dynamic blend that provided daily ratio of SID Lys-to-NE to optimize milk nitrogen output for second parity sows throughout a 21-day lactation period.

Two isocaloric basal diets with SID Lys-to-NE ratios of 2.85 and 5.50 g SID Lys/Mcal NE (Table 1) were blended in varying proportions to achieve one of two feeding programs: [1] a control feeding program that consisted of a static blend of the two diets to deliver 3.91 g SID Lys/ Mcal NE throughout the entirety of lactation for both first and second parity sows (**CON**; Figure 1) or [2] precision feeding programs using a dynamic blend of the basal diets to meet estimated daily optimal SID Lys-to-NE ratios throughout lactation (**PRE**). The PRE feeding programs were created to optimize maternal nitrogen retention for first parity sows (ranging from 5.48 to 4.95 g SID Lys/Mcal NE on days 1 and 20, respectively; Gregory and Huber, 2025 ; Figure 2) and to optimize milk nitrogen output for second parity sows (ranging from 3.12 to 4.68 g SID Lys/Mcal NE on days 1 and 20, respectively; Watzeck and Huber, 2024; Figure 3). In the aforementioned studies, only maternal nitrogen retention or milk nitrogen output responded to SID Lys-to-NE ratio in lactation for primiparous and multiparous sows, respectively. Thus, a 2 × 2 factorial design was created with feeding program (CON vs. PRE) and parity (1 vs. 2) as the factors (n = 25 or 26; Parity 1-CON: 26; Parity 2-CON: 25; Parity 1-PRE: 26; Parity 2-PRE: 26).

**Table 1.** Ingredient and nutrient composition of basal diets (as-fed)

	SID Lys: NE, g/Mcal^1^	
	2.85	5.50
Ingredient, %		
Corn	45.57	33.89
Wheat, soft red	20.00	25.00
Soybeans, full fat	11.00	20.00
Soybean meal	10.00	15.50
Soybean hulls	6.50	0
Fat, animal-vegetable blend	2.85	0.05
Monocalcium phosphate	1.40	1.25
Limestone, ground	1.30	1.35
Premix^2^	0.60	0.60
Sodium chloride	0.50	0.50
L-Lys-HCL	0.12	0.53
L-Val	0.08	0.38
L-Thr	0.08	0.24
L-Leu	0	0.21
DL-Met	0	0.15
L-Phe	0	0.15
L-His-HCL	0	0.11
L-Trp	0	0.05
L-Ile	0	0.04
Total	100.00	100.00
Calculated nutrient contents^3^		
Net energy, kcal/kg	2,543	2,542
Crude protein, %	15.74	23.25
SID Lys, %	0.72	1.40
SID Met, %	0.22	0.44
SID Thr, %	0.53	0.89
SID Leu, %	1.08	1.62
SID Val, %	0.66	1.2
SID Ile, %	0.51	0.79
SID His, %	0.35	0.56
SID Phe, %	0.63	1.03
Total Calcium, %	0.90	0.92
STTD P, %^4^	0.45	0.46
Fermentable fiber, %	11.17	11.98
Analyzed nutrient contents^5^		
Crude Protein, %	15.01	23.13
Lys, %	0.78 (0.86)^6^	1.43 (1.59)

^1^Standardized ileal digestible lysine-to-net energy ratio; basal diets were blended in varying proportions during lactation.

^2^Provided per kg of premix: Calcium 22.67% as CaCO_3_; Manganese, 4,000 mg as MnSO_4_ H_2_O; Zinc, 21,000 mg as ZnSO_4_; Iron, 20,000 mg as FeSO_4_; Copper, 3,000 mg as CuSO_4_; Selenium, 60 mg as Na_2_SeO_3_; Iodine, 100 mg as C_2_H_10_I_2_H_2_; Vitamin A, 2,000 KIU; Vitamin D, 200 KIU; Vitamin E, 8,000 IU; Vitamin K, 500 mg; Thiamin, 200 mg; Riboflavin, 1,000 mg; Niacin, 5,000 mg; Panthothenic Acid, 3,000 mg; Pyridoxine, 300 mg; Choline, 100,000 mg; Folacin, 400 mg; Biotin, 40 mg; Vitamin B_12_, 5,000 mcg (Grand Valley Fortifiers Ltd., Cambridge, ON, Canada).

^3^Based on standardized ileal digestible nutrient and net energy contents of feed ingredients according to the NRC (2012).

^4^Standardized total tract digestible.

^5^Analyzed values for a composite sub-sample of seven batches per diet.

^6^Calculated values are shown in parentheses.

### Animal Sampling

Sow BW was measured after farrowing, day 10 of lactation, and at weaning and backfat depth (**BF**) was measured via ultrasound (Honda Electric Co. Ltd, Toyohashi, Aichi, Japan) at the P2 position at farrowing and weaning. The amount of feed dispensed to each sow was recorded daily using the electronic feeding system, which was calibrated weekly, and feed refusals were measured daily to determine average daily feed intake (**ADFI**). Individual piglet BW were measured at birth, days 7 and 14 of lactation, and at weaning. A subsample of 12 sows per feeding program and parity combination were randomly selected on day 1 of lactation and had blood samples collected after a 17-hour fast on days 2, 10, and at weaning via an orbital sinus puncture. Blood was collected in a 10-mL untreated serum tube and a 6-mL sodium heparin tube (BD vacutainer, Franklin Lakes, NJ). Serum was centrifuged at room temperature and plasma was centrifuged at 4 °C, both for 15 min at 1,500 × g. Serum and plasma samples were aliquoted and stored at -20 °C until further analysis.

### Analysis

Serum was analyzed for beta-hydroxy butyric acid (**BHBA**), non-esterified fatty acids (**NEFA**), and urea at the University of Guelph Animal Health Laboratories (Guelph, ON, Canada) using a Conas 600 c501 biochemistry analyzer (Rocher Diagnosis, Laval, QC, Canada). Plasma amino acids (**AA**) were analyzed via ultra-performance liquid chromatography adapted from [(Bidlingmeyer et al., 1984); Waters Corporation, Milford, MA] as described by Banton et al. (2021). Derivatization was completed with the AccQ-Tag Ultra derivatization kit (Waters Corporation, Milford, MA). Peak areas for AA were compared to the peaks of known standards and were analyzed using Waters Empower 2 Software (Waters Corporation, Milford, MA). Feed AA contents were analyzed via oxidative hydrolysis, with feed samples derivatized and analyzed following a similar protocol to plasma AA.

### Statistical Analysis

The analysis of variance was completed using the PROC GLIMMIX function of SAS 9.4. The fixed effects of feeding program (CON vs. PRE), parity (1 vs. 2), and the interaction between feeding program and parity and the random effect of block were included in the model. Sow (or litter) was the experimental unit. Mean comparisons were conducted using the Tukey Kramer post-hoc test when the interaction was significant. The serum metabolites BHBA, urea, and the plasma amino acids were log transformed and then back transformed to present the means. Probability (***P***) values less than 0.05 were considered significant, while 0.05 ≤ *P* ≤ 0.10 were considered a trend. Any *P*-values greater than 0.10 were considered not significant.

## RESULTS

Analyzed nutrient values for both basal diets (2.85 and 5.50 g SID Lys:Mcal NE) were comparable to the calculated nutrient contents. Three sows were removed from study due to illness (1 × Parity 1-CON, 1 × Parity 1-PRE, 1 × Parity 2-PRE) and two were removed due to low feed intake (≥ 3 days with 60% or less of allotted feed consumed; 1 × Parity 1-CON and 1 × Parity 1-PRE); data from these sows were not included in the statistical analysis.

### Growth Performance

Sow initial BW and BF were not influenced by feeding program or the interaction between parity and feeding program, however second parity sows had greater initial and weaning BW, initial BF, and less BW and BF loss during lactation than first parity sows (Parity; *P* < 0.05; Table 2). Sows fed the PRE program tended to lose less BF throughout lactation compared to sows fed the CON program, regardless of parity (Feeding program; *P* = 0.094). No differences were observed for ADFI between feeding programs, however second parity sows consumed more feed than first parity sows (Parity; *P* < 0.001) and ADFI tended to be influenced by the interaction between feeding program and parity (*P *= 0.053), but no differences were detected during means separation.

**Table 2.** Performance of first and second parity sows fed either a static (CON) or dynamic (PRE) blend of the basal diets throughout a 21-day lactation period

	CON		PRE^1^		SEM^2^	*P*-value^3^		
Item	Parity 1	Parity 2	Parity 1	Parity 2		Trmt	Parity	Trmt × Parity
No.^4^	24	25	24	25				
Average daily feed intake, kg	5.39	6.06	5.25	6.33	0.10	0.552	< 0.001	0.053
Sow BW, kg								
Initial	196.8	205.9	197.8	203.7	3.6	0.823	0.008	0.553
Weaning^5^	183.4	202.4	186.4	200.8	3.4	0.807	< 0.001	0.406
Total loss	−13.3	−3.7	−11.1	−3.1	3.2	0.442	< 0.001	0.667
Sow back fat depth, mm								
Initial	12.9	11.3	12.6	10.3	0.4	0.110	< 0.001	0.490
Weaning^5^	9.5	9.3	9.6	8.7	0.4	0.476	0.139	0.301
Total loss	−3.4	−2.1	−2.9	−1.8	0.5	0.094	< 0.001	0.735

^1^The CON sows received a static blend of low and high standardized ileal digestible (SID) Lys-to-net energy (NE) ratio diets whereas PRE sows received a dynamic blend of the basal diets to achieve the estimated daily optimal SID Lys-to-NE ratio throughout the lactation period.

^2^Standard error of means.

^3^Probability of main effects of dietary treatment (Trmt), parity, and the interaction of dietary treatment and parity.

^5^Number of animals evaluated.

^6^Weaning occurred on day 20 ± 0.4 of lactation.

### Piglet Performance

The number of piglets born alive, stillborn, and mummified were not influenced by feeding program, parity, or the interaction between feeding program and parity (Table 3). Litter birth weight was not different between feeding programs but was greater for first versus second parity sows (Parity; *P *< 0.005). Initial litter size (after standardization), litter size at weaning, piglet average daily gain (**ADG**), and litter gain during week 1 of lactation were not influenced by feeding program, parity, or the interaction between feeding program and parity. Piglet BW at birth, day 7, and day 14 of age were not influenced by feeding program or the interaction between feeding program and parity, while piglets from second parity sows were heavier at birth, day 14, and at weaning with greater piglet ADG and litter gain in week 2 and overall than piglets from first parity sows (Parity; *P* < 0.05). During week 3 and overall, piglets from sows that received the PRE feeding program had greater piglet ADG and litter gain compared to those from sows on the CON program corresponding to greater piglet BW at weaning for PRE-fed sows, regardless of parity (Feeding program; *P* < 0.05).

**Table 3.** Growth performance of offspring from first parity and second parity sows fed either a static (CON) or dynamic (PRE) blend of the basal diets throughout a 21-day lactation period

	CON		PRE^1^		SEM^2^	*P*-value^3^		
Item	Parity 1	Parity 2	Parity 1	Parity 2		Trmt	Parity	Trmt × Parity
No.^4^	24	25	24	25				
Piglets born alive, no.	14.7	14.3	13.8	14.5	0.6	0.495	0.809	0.346
Stillborn, no.	0.9	0.8	0.4	0.9	0.2	0.369	0.286	0.273
Mummified, no.	0.2	0.3	0.5	0.2	0.1	0.367	0.398	0.150
Litter birth weight, kg^5^	21.1	22.5	20.5	23.3	0.9	0.946	0.004	0.308
Litter size initial^6^	13.6	13.4	13.5	13.3	0.4	0.566	0.516	0.961
Litter size weaning^7^	13.2	13.0	12.6	13.0	0.4	0.181	0.677	0.275
Piglet BW, kg								
Birth	1.48	1.55	1.50	1.62	0.05	0.254	0.038	0.616
Day 7	2.81	2.79	2.81	2.95	0.12	0.206	0.396	0.220
Day 14	4.52	4.72	4.66	4.88	0.15	0.127	0.033	0.936
Wean	6.11	6.27	6.25	6.64	0.17	0.017	0.012	0.285
Piglet average daily gain, g								
Week 1	186	174	185	185	15	0.564	0.430	0.415
Week 2	244	276	262	280	10	0.231	0.007	0.426
Week 3	255	253	271	288	17	0.008	0.424	0.294
Overall	229	234	237	248	9	0.022	0.104	0.546
Litter gain, kg								
Week 1	16.5	15.5	16.6	16.4	1.1	0.500	0.357	0.578
Week 2	22.4	24.3	23.0	24.9	1.1	0.458	0.018	0.998
Week 3	19.2	19.3	20.9	22.2	1.5	0.003	0.340	0.430
Overall	58.3	59.4	60.4	63.2	2.3	0.024	0.152	0.524

^1^The CON sows received a static blend of low and high standardized ileal digestible (SID) Lys-to-net energy (NE) ratio diets whereas PRE sows received a dynamic blend of the basal diets to achieve the estimated daily optimal SID Lys-to-NE ratio throughout the lactation period.

^2^Standard error of means.

^3^Probability of main effects of dietary treatment (Trmt), parity, and the interaction of dietary treatment and parity.

^4^Number of litters evaluated.

^5^Includes stillborn piglets; data collected prior to implementing experimental feeding programs.

^6^After litter standardization.

^7^Weaning occurred on day 20 ± 0.4 of lactation.

### Blood Metabolites

On day two of lactation, feeding program, parity, and the interaction between feeding program and parity did not influence serum concentrations of BHBA and urea (Table 4). Second parity sows had lower serum NEFA concentrations compared to first parity sows (Parity; *P* < 0.05) and sows fed the PRE programs had lower serum NEFA concentrations than sows fed the CON program, regardless of parity (Feeding program; *P* < 0.05). On day 10 of lactation, feeding program, parity, and the interaction between feeding program and parity did not influence serum BHBA and NEFA concentrations. Serum urea concentration was greater for sows fed the PRE program compared to the CON program (Feeding program; *P* < 0.05), regardless of parity. At weaning, serum concentrations of BHBA and urea were greater for sows fed the PRE programs compared sows fed CON (Feeding program; *P* < 0.05), regardless of parity, and serum NEFA concentrations were not influenced by feeding program, parity, or the interaction between feeding program and parity.

**Table 4.** Post-absorptive serum concentrations of beta-hydroxybutyric acid (BHBA), non-esterified fatty acids (NEFA), and urea for first and second parity sows fed either a static (CON) or dynamic (PRE) blend of the basal diets throughout a 21-day lactation period

	CON		PRE^1^		SEM^2^	*P*-value^3^		
Item	Parity 1	Parity 2	Parity 1	Parity 2		Trmt	Parity	Trmt × Parity
No.^4^	12	12	12	12				
BHBA, μmol/L								
Day 2	14.6	20.3	18.2	16.5	2.6	0.971	0.526	0.397
Day 10	4.6	7.9	7.4	10.7	3.4	0.385	0.365	0.995
Weaning^5^	25.8	34.4	39.7	42.1	23.2	0.022	0.231	0.497
NEFA, mmol/L								
Day 2	0.72	0.45	0.52	0.25	0.08	0.009	0.002	0.967
Day 10	0.37	0.29	0.42	0.41	0.17	0.380	0.581	0.587
Weaning^5^	0.74	0.58	0.98	0.68	0.31	0.272	0.126	0.729
Urea, mmol/L								
Day 2	3.3	3.7	4.0	3.9	0.4	0.244	0.690	0.568
Day 10	5.7	5.7	7.3	6.4	0.3	< 0.001	0.155	0.110
Weaning^5^	5.3	5.5	6.6	7.0	0.4	< 0.001	0.292	0.652

^1^The CON sows received a static blend of low and high standardized ileal digestible (SID) Lys-to-net energy (NE) ratio diets whereas PRE sows received a dynamic blend of the basal diets to achieve the estimated daily optimal SID Lys-to-NE ratio throughout the lactation period.

^2^Standard error of means.

^3^Probability of main effects of dietary treatment (Trmt), parity, and the interaction of dietary treatment and parity.

^4^Number of animals evaluated.

^5^Weaning occurred on day 20 ± 0.4 of lactation.

### Plasma Free Amino Acid Concentrations

Plasma concentrations of Arg, Met, or Phe were not influenced by feeding program, parity, or the interaction between feeding program and parity at any point in lactation (Table 5). On day two of lactation, the main effect of feeding program did not influence plasma AA concentrations. Plasma Val concentrations were less for second parity versus first parity sows only when receiving the PRE program (Interaction; *P* < 0.05), while Leu and Asp tended to be influenced by the interaction between feeding program and parity (*P* = 0.065 and 0.071, respectively). Plasma Trp concentrations were less and plasma concentrations of Ala, Gln, and Pro were greater for second parity sows compared to first parity sows (Parity; *P* < 0.05). On day 10 of lactation, plasma Glu concentrations were greater for second parity versus first parity sows only when receiving the PRE program (Interaction; *P* < 0.05). Plasma concentrations of Lys, Thr, Val, and total essential amino acids (**EAA**) were greater for sows fed the PRE program versus CON (Feeding program; *P* < 0.05), while Ile tended to be greater and Ala tended to be less for PRE program-fed sows (Feeding program; *P* = 0.096 and *P *= 0.098, respectively). Second parity sows had lower concentrations of His, Lys, Thr, Trp, Val, total EAA, and Tyr compared to first parity sows, while Asp concentrations were greater for second compared to first parity sows (Parity; *P* < 0.05). Plasma Gly concentration tended to be greater and Ile tended to be less for second parity sows compared to first parity sows (Parity; *P* = 0.061 and *P* = 0.088, respectively). At weaning, plasma concentrations of Tyr, Ser, Glu, Asp, Asn, and total non-essential amino acids (**NEAA**) were lower for sows fed PRE programs compared to sows fed the CON program (Feeding program; *P* < 0.05), while Trp and Ala tended to be less for PRE (Feeding program; *P* = 0.093 and *P* = 0.089). Second parity sows had greater plasma concentrations of Glu, Ala, and total NEAA compared to first parity sows (Parity; *P* < 0.05), while Ser and Gly tended to be greater for second versus first parity sows (Parity; *P* = 0.092 and *P* = 0.060).

**Table 5.** Post-absorptive plasma free essential (EAA) and nonessential (NEAA) amino acids (μmol/L) from first and second parity sows fed either a static (CON) or dynamic (PRE) blend of the basal diets throughout a 21-day lactation period

	CON		PRE^1^		SEM^2^	*P*-value^3^		
Item	Parity 1	Parity 2	Parity 1	Parity 2		Trmt	Parity	Trmt × Parity
No.^4^	12	12	12	12				
Arg								
Day 2	122	134	131	127	12	0.919	0.736	0.443
Day 10	85	89	103	89	11	0.184	0.465	0.182
Weaning^5^	82	83	72	77	10	0.150	0.629	0.738
His								
Day 2	105	96	103	101	4	0.638	0.233	0.375
Day 10	117	102	114	103	6	0.855	0.030	0.709
Weaning	86	83	78	80	5	0.144	0.947	0.424
Ile								
Day 2	104	108	115	101	6	0.744	0.384	0.118
Day 10	88	77	96	88	7	0.096	0.088	0.774
Weaning	96	93	103	106	16	0.141	0.974	0.634
Leu								
Day 2	138	147	165	139	11	0.318	0.420	0.065
Day 10	156	136	151	152	13	0.588	0.333	0.326
Weaning	166	168	162	180	20	0.681	0.241	0.353
Lys								
Day 2	153	177	177	140	17	0.758	0.734	0.329
Day 10	93	82	130	88	14	0.022	0.004	0.126
Weaning	101	93	109	98	20	0.461	0.287	0.883
Met								
Day 2	42	43	42	40	3	0.574	0.952	0.667
Day 10	34	32	40	35	4	0.103	0.175	0.585
Weaning	29	28	25	28	3	0.264	0.542	0.378
Phe								
Day 2	64	58	67	60	5	0.569	0.126	0.867
Day 10	63	55	65	61	7	0.356	0.183	0.651
Weaning	71	69	61	60	14	0.146	0.817	0.943
Thr								
Day 2	150	167	141	140	16	0.221	0.574	0.549
Day 10	77	62	106	70	15	0.016	0.001	0.266
Weaning	83	84	84	70	13	0.250	0.290	0.234
Trp								
Day 2	38	30	36	27	3	0.301	0.008	0.859
Day 10	37	30	43	33	5	0.136	0.014	0.744
Weaning	47	43	40	39	6	0.089	0.415	0.606
Val								
Day 2	250^b^	250^b^	326^a^	222^b^	15	0.105	0.002	0.001
Day 10	241	227	308	240	24	0.028	0.019	0.161
Weaning	255	221	245	255	21	0.424	0.422	0.153
Total EAA								
Day 2	1177	1260	1310	1113	67	0.939	0.556	0.307
Day 10	993	907	1184	966	89	0.027	0.006	0.280
Weaning	1037	995	990	1027	109	0.869	0.943	0.398
Ala								
Day 2	519	573	501	628	46	0.704	0.029	0.379
Day 10	406	390	321	373	36	0.098	0.498	0.270
Weaning	281	294	221	290	33	0.093	0.036	0.133
Asn								
Day 2	53	56	62	59	5	0.164	0.985	0.466
Day 10	59	55	59	53	7	0.763	0.312	0.804
Weaning	49	52	40	46	6	0.038	0.164	0.590
Asp								
Day 2	16	19	18	18	1	0.626	0.189	0.071
Day 10	16	17	14	17	1	0.378	0.044	0.187
Weaning	13	14	12	12	1	0.039	0.495	0.834
Cys								
Day 2	22	25	20	22	4	0.424	0.424	0.882
Day 10	11	11	11	13	2	0.810	0.548	0.610
Weaning	8	7	6	10	2	0.992	0.294	0.047
Gln								
Day 2	411	461	430	499	60	0.270	0.022	0.757
Day 10	406	416	375	376	76	0.301	0.876	0.894
Weaning	308	307	245	282	53	0.129	0.495	0.485
Glu								
Day 2	173	193	178	186	16	0.926	0.264	0.588
Day 10	189^ab^	190^a^	149^b^	198^a^	21	0.128	0.024	0.036
Weaning	134	159	119	134	31	0.004	0.004	0.559
Gly								
Day 2	876	827	830	818	67	0.676	0.643	0.773
Day 10	914	933	773	974	65	0.347	0.061	0.116
Weaning	773	809	652	776	120	0.072	0.060	0.268
Pro								
Day 2	218	251	222	265	17	0.621	0.034	0.812
Day 10	209	210	184	216	20	0.507	0.280	0.313
Weaning	157	160	137	162	17	0.341	0.139	0.220
Ser								
Day 2	114	114	118	111	6	0.884	0.407	0.449
Day 10	110	111	105	116	5	0.990	0.145	0.212
Weaning	114	116	96	110	8	0.020	0.092	0.178
Tyr								
Day 2	62	62	68	64	5	0.364	0.692	0.732
Day 10	72	58	73	66	6	0.300	0.041	0.462
Weaning	60	63	49	54	4	0.009	0.241	0.747
Total NEAA								
Day 2	2496	2598	2489	2721	128	0.665	0.206	0.624
Day 10	2410	2411	2110	2432	168	0.307	0.229	0.249
Weaning	1922	1994	1596	1902	228	0.020	0.033	0.159

^1^The CON sows received a static blend of low and high standardized ileal digestible (SID) Lys-to-net energy (NE) ratio diets whereas PRE sows received a dynamic blend of the basal diets to achieve the estimated daily optimal SID Lys-to-NE ratio throughout the lactation period.

^2^Standard error of means.

^3^Probability of main effects of dietary treatment (Trmt), parity, and the interaction of dietary treatment and parity.

^4^Number of animals evaluated.

^5^Weaning occurred on day 20 ± 0.4 of lactation.

## DISCUSSION

The objective of the current study was to evaluate the effects of feeding daily optimal SID Lys-to-NE ratios for first and second parity lactating sows on piglet growth performance and sow body mobilization. The results demonstrated that feeding unique and dynamic SID Lys-to-NE ratios to first and second parity sows benefited piglet growth, particularly in the third week of lactation. It has previously been shown that piglet growth is limited by milk output by day 10 of lactation, thus, greater piglet growth rates during peak lactation implies increased milk yield and/or energy density of the milk (Theil et al., 2012). Both feeding programs provided sows with similar feed (energy) intakes (5.7 versus 5.8 ± 0.1 kg/d for CON and PRE sows, respectively; average of first and second parity sows within feeding program) but greater SID Lys intake during the week of peak lactation for PRE-fed sows (days 14 to 21; first parity sows received 72 and 58 g SID Lys per day for PRE and CON, respectively; second parity sows received 84 and 67 g SID Lys per day for PRE and CON, respectively). Thus, at similar energy intakes, altering the supply of SID Lys promoted piglet growth in peak lactation, lending further evidence that the optimal dietary SID Lys-to-NE ratio is related to milk production (Feyera and Theil, 2017), at least for first and second parity sows. Pedersen et al. (2016) showed that increasing SID Lys and energy supply in the last 2 wk of lactation for second parity sows tended to improve piglet BW at weaning. Previous work has also found that piglet growth performance responded linearly to increasing Lys intake throughout the entirety of lactation, primarily for young, immature sows (average 52 to 78 g total Lys intake per day; Greiner et al., 2020). Additionally, Hojgaard et al. (2019a) found a linear improvement for piglet ADG when sows were fed increasing levels of SID Lys from 40 and 63 g/d throughout lactation for both primiparous and multiparous sows. Therefore, feeding programs that provide greater Lys intakes, and therefore Lys-to-NE ratios, can improve milk production and support piglet growth, especially in peak lactation.

In the current study, there were no differences in sow BW at weaning or total BW loss between the feeding programs, while second parity sows were heavier and lost less BW over the course of the 21-day lactation period. In Gregory and Huber (2025), altering the SID Lys-to-NE ratio for first parity sows did not affect piglet ADG or piglet BW at weaning, but instead, differences were observed solely in the maternal body (maternal nitrogen retention and changes in BW and BF), which was not replicated in the current study. Thus, the lack of difference for sow BW loss between the two feeding programs was unexpected, for first parity sows in particular, since the first parity PRE feeding program was designed to optimize maternal nitrogen retention (Gregory and Huber, 2025). Despite the comparable ADFI, sow BW loss was relatively greater, which could have contributed to the 12% greater piglet ADG observed in the current study versus that of the primiparous sows in Gregory and Huber (2025). It has been shown previously that when milk yield increased by 1 kg per day, sow BW loss can increase by at least 4.3 kg, assuming no change in ADFI (Strathe et al., 2017). Additionally, in the current study, total BF loss tended to be reduced for sows that received the PRE feeding program, regardless of parity, which aligns Gregory and Huber (2025) where first parity sows had reduced BF loss as the SID Lys-to-NE ratio increased. Therefore, providing a dynamic SID Lys-to-NE ratio to both first and second parity sows can be a means to improve overall piglet growth performance in the suckling period, without increasing maternal body mobilization.

In the current study, the PRE feeding program for multiparous sows was created using SID Lys-to-NE ratios that maximized piglet growth rates and weekly milk nitrogen output for sows with an average parity of 3.1 ± 0.9 (Watzeck and Huber, 2024). In the current study, however, second parity sows were the oldest females available at the research facilities and therefore, were not as mature and had relatively lower BW compared to the multiparous sows used in Watzeck and Huber (2024). Moreover, second parity sows generally begin lactation with lower BW and have inferior feed intake, which can result in greater BW loss in lactation compared to older sows (Stewart et al., 2021; Estrada et al., 2024). Therefore, using second parity versus third parity sows in the current study could have affected the magnitude of responses like piglet growth performance or sow BW loss, but it is posited that since older sows also follow a milk production curve, providing dynamic SID Lys-to-NE ratios throughout lactation would also benefit sows in the third parity and beyond.

During the second day of lactation, fasted serum concentrations of NEFA were lower for sows that received the PRE relative to the CON feeding program, suggesting less mobilization of fat stores at the beginning of lactation. Despite only receiving the respective feeding program for 1 d prior to blood sampling, sows on the PRE programs consumed 25% more feed within the first 24 hours after farrowing regardless of parity (2.5 versus 1.9 ± 0.1 kg for PRE and CON, respectively; data not shown), which could have allowed the PRE-fed sows to better meet the energy demands of lactation. Thus, greater feed intake during the first day of lactation can reduce mobilization from maternal fat stores. Additionally, on lactation day 10 and at weaning, PRE-fed sows had greater fasted serum urea concentrations compared to sows that received the CON program, which was attributed to greater daily intakes of crude protein due to higher inclusion of the 5.50 g SID Lys/Mcal NE basal diet in the blend. Indeed, 55% of the additional SID Lys was supplied by soybean products in the 5.50 g SID Lys/Mcal NE diet, which was necessary to achieve all recommended essential AA:Lys ratios, while not excessively relying on crystalline AA. Such an increase in serum urea has been observed in other studies where sows were fed greater levels of crude protein during lactation (Hojgaard et al., 2019b; Liu et al., 2020) and is likely due to deamination and oxidation of excess AA. Since there was no difference in sow BW loss during lactation, the greater serum urea concentrations were likely due to feed composition rather than AA mobilized from maternal body protein. Moreover, at weaning, PRE-fed sows had lower plasma NEAA concentrations compared to CON, specifically for gluconeogenic AA precursors like Ser, Gly, and Gln, indicating that PRE-fed sows were relying less on body protein mobilization in peak lactation (Reynolds et al., 1994). Therefore, during instances of high energy and Lys demand (e.g., peak lactation), the greater SID Lys-to-NE ratios provided in a dynamic feeding program likely allowed for better maintenance of the maternal protein pool for both first and second parity sows.

At weaning, elevated serum concentrations of BHBA were observed for sows that received the PRE versus CON feeding program. Since BHBA is a ketone produced by the break-down of body fat stores when in an energy-restricted state (Theil et al., 2013), sows on the PRE feeding program could have been mobilizing additional body fat stores by weaning, which contradicts the observation of PRE-fed sows tending to lose less BF over lactation compared to CON-fed sows. As previously mentioned, piglets from PRE-fed sows had greater ADG specifically during the third week of lactation, indicating greater milk production and/or greater concentrations of milk energetic components. Thus, it is possible that in peak lactation, mobilization of maternal body fat stores was increased for PRE-fed sows, but was not detectable as differences in BF thickness and/or fat was mobilized from other body pools. Future research should consider an extended lactation period for sows and assess feeding strategies that support increased milk production for prolonged periods, while minimizing maternal body tissue mobilization.

Finally, the PRE feeding programs used in the current study required the use of feeding systems with feed blending capabilities and relied to a greater extent on the more expensive 5.5 g SID Lys/Mcal NE basal diet in the daily blends (approximately 6% and 2% greater feed costs versus CON for first and second parity sows, respectively; data not shown). The improvements in piglet body weight at weaning from PRE-fed sows could help to mitigate equipment and feed costs, but return on investment would be system-specific.

## CONCLUSION

In conclusion, providing first and second parity sows a dynamic feeding program throughout lactation to meet estimated daily optimal SID Lys-to-NE ratios improved piglet growth rate in peak lactation and BW at weaning, without increasing BW loss and BF mobilization of the sows. Thus, the implementation of feeding programs that optimize the supply of both energy and SID Lys throughout lactation has the potential to improve performance of lactating sows, particularly when milk production is high. Therefore, a dynamic feeding program could provide an additional benefit for farms with access to electronic sow feeders with feed blending capabilities in the farrowing room.
